# Protocol to analyze and validate transcriptomic changes in PDGFRβ-KO mesenchymal stem cell osteogenic potential in the mouse embryo

**DOI:** 10.1016/j.xpro.2022.102016

**Published:** 2023-01-12

**Authors:** Alastair Morris Kilpatrick, Madalena Marques, Mario Gomez-Salazar, Dorota Stefancova, Diana Sá da Bandeira, Mihaela Crisan

**Affiliations:** 1 Centre for Regenerative Medicine, Institute for Regeneration and Repair, University of Edinburgh, 5 Little France Drive, Edinburgh EH16 4UU, UK; 2 Centre for Cardiovascular Science, The Queen’s Medical Research Institute, University of Edinburgh, Edinburgh EH16 4TJ, UK

**Keywords:** Bioinformatics, Cell Biology, Single Cell, Developmental Biology, RNAseq, Molecular Biology, Gene Expression, Stem Cells, Cell Differentiation

## Abstract

Mesenchymal stem/stromal cells (MSCs) can differentiate into osteoblasts under appropriate conditions. PDGFRβ signaling controls MSC osteogenic potential both transcriptomically and in culture. Here, we present a “computer to the bench” protocol to analyze changes in MSC osteogenic potential at transcriptomic and cellular level in the absence of PDGFRβ. We detail the preparation of cells from mouse embryos, the analysis of transcriptomic changes from single-cell RNA-sequencing data, the procedure for MSC derivation and culture, and an osteogenic assay for functional validation.

For complete details on the use and execution of this protocol, please refer to Sá da Bandeira et al. (2022).^1^

## Highlights

• Aorta-gonad-mesonephros region (AGM) cell preparation and genotyping prior to analysis
• Single-cell RNA sequencing to analyze transcriptomic changes in PDGFRβ-KO MSCs
• Derivation and expansion of PDGFRβ-WT and KO AGM MSCs in similar conditions *in vitro*
• An osteogenic assay to confirm altered KO MSC osteogenic potential *in vitro*

## Abstract (editor highlights)

Publisher’s note: Undertaking any experimental protocol requires adherence to local institutional guidelines for laboratory safety and ethics.

## Graphical abstract

**Figure:**
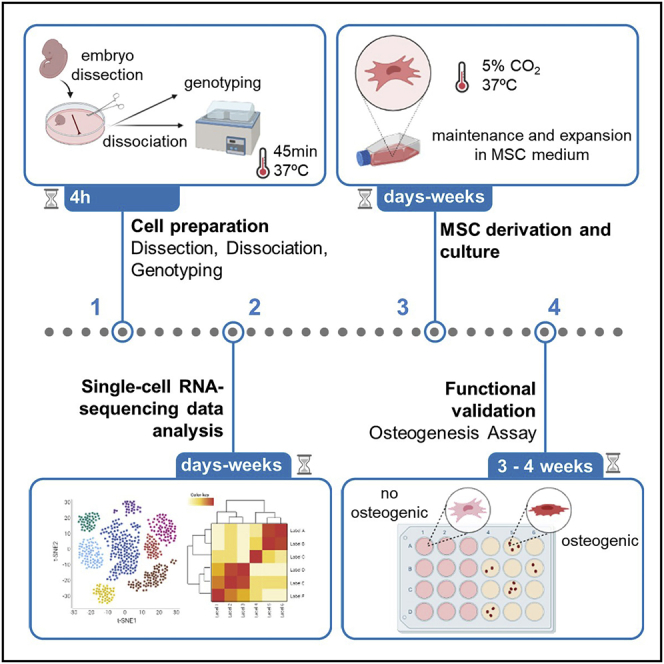

## Before you begin

In mice, the first adult-type hematopoietic stem cells (HSCs) arise from hemogenic endothelial cells lining the dorsal aorta in the aorta-gonad-mesonephros region (AGM) at developmental day (E) 10.5.^2^ Endothelial cells are surrounded by mesenchymal perivascular cells expressing PDGFRβ that were recently shown to support hematopoietic stem and progenitor cell (HSPC) development.^1^ Indeed, the AGM HSPC number was significantly reduced in the absence of PDGFRβ. Moreover, upon culture, we found that mesenchymal stem/stromal cells (MSCs) derived from PDGFRβ-KO AGMs showed reduced hematopoietic support in co-culture experiments and had low ability to differentiate towards bone.

The following protocol describes detailed steps to analyze MSCs from unfractionated E11 AGMs *in vivo* by single-cell RNA sequencing and proposes a novel method to validate transcriptomic changes in PDGFRβ-KO MSCs in single AGM-derived cell culture. Here, we modified a culture method described previously.^3^^,^^4^ We derived MSCs from single AGMs to study PDGFRβ-WT (+/+) and PDGFRβ-KO (−/−) embryos individually, and we investigated their bone forming potential at higher scale.^1^

### Institutional permissions

All experiments were performed under a Project License granted by the Home Office UK, approved by the University of Edinburgh Ethical Review Committee (70-8417/05-02-2015; 70-8568/24-04-2017; PP8962771/23-10-2020) and conducted in accordance with local guidelines.

### Hardware

1. Access to a 64-bit Linux system with a 16-core Intel or AMD processor, 128 GB memory, and 1TB disk space is required. To run 10× Cell Ranger in cluster mode, the cluster requires a shared file system (e.g., NFS) and a batch scheduling system (e.g., SGE).
2. Required local hardware: we suggest a minimum of 8 GB memory and 10 GB local disk storage for downstream analysis.

### Software

3. R/Bioconductor installation. Required packages are listed in the key resources table (KRT).

Recommended: RStudio IDE.

4. The mouse reference dataset, required for 10× Cell Ranger, can be downloaded from

https://support.10xgenomics.com/single-cell-gene-expression/software/downloads/latest.

5. The R software environment can be downloaded from https://www.r-project.org/. RStudio Desktop is highly recommended as an integrated development environment for R and can be downloaded from https://www.rstudio.com/products/rstudio/.

If required, download and install Bioconductor from within RStudio as follows:

>if (!require("BiocManager", quietly = TRUE))

 install.packages("BiocManager")

>BiocManager::install(version = "3.15")

Additional R/Bioconductor packages can be downloaded and installed via:

>install.packages("package_name")

or

>BiocManager::install("package_name")

as appropriate.

## Key resources table

**Table:** 

REAGENT or RESOURCE	SOURCE	IDENTIFIER
**Biological samples**		

E11 AGM from PDGRFβ+/+ and PDGRFβ−/−	N/A	N/A

**Chemicals, peptides, and recombinant proteins**		

DPBS -Ca/-Mg	Sigma	Cat# D8537
Fetal calf serum (FCS)	Life Tech	Cat# 10270106
Penicillin/streptomycin (PS)	Invitrogen	Cat# 15140-122
Collagenase type I	Sigma	Cat# C0130
Paraformaldehyde (PFA)	VWR International	Cat# J61899
DNA extraction kit (extraction solution)	Sigma	Cat# E7526
DNA extraction kit (tissue preparation solution)	Sigma	Cat# T3073
DNA extraction kit (neutralization solution)	Sigma	Cat# N3910
Hot Start Taq Plus Kit	Qiagen	Cat# 203605
Agarose	Bioline	Cat# 41025
10× TAE buffer	Invitrogen	Cat# 15558-026
SYBR®Safe DNA Gel stain	Invitrogen	Cat# 533102
EasyLadder I	Bioline	Cat# 33046
100 mM dNTP Set	Invitrogen	Cat# 10297-018
Gelatin	Sigma	Cat# G1890
Myelocult	Stem Cell Technology	Cat# 05350
αMEM	Gibco	Cat# 22571-020
Glutamax 100×	Gibco	Cat# 35050-038
β-Mercaptoethanol	Life Tech	Cat# 31350-010
Dimethyl sulfoxide (DMSO)	VWR Chemicals	Cat# 23500.260
Dexamethasone	Sigma	Cat# D4902
L-Ascorbic acid-2-phosphate (L-AA)	Sigma	Cat# A8960
β-Glycerophosphate disodium pentahydrate (β-GP)	Calbiochem	Cat# 35675
DMEM With glutamax	Gibco	Cat# 61965026
Alizarin Red S	Sigma	Cat# A5533-25G
0.25% Trypsin + EDTA	Life Tech	Cat# 25200-072
Trypan Blue solution 0.4%	Sigma	Cat#T8154

**Deposited data**		

Single-cell RNA seq	Sá da Bandeira et al.^1^	NCBI GEO Accession number: GSE162103
Mouse reference genome (mm10/GRCm38–3.0.0)	10x Genomics	https://support.10xgenomics.com/single-cell-gene-expression/software/downloads/latest

**Experimental models: Cell lines**		

PDGFRβ KO AGM MSCs	Sá da Bandeira et al.^1^	N/A

**Experimental models: Organisms/strains**		

Mice: 3–6 month old, males and females PDGFRβ KO	Christer Betsholtz	Soriano^5^

**Software and algorithms**		

Zen Lite (v2.6) and Pro (v2.3) softwares	Zeiss	www.zeiss.com/microscopy/int/products/microscope-software/zen.html
Fiji/ImageJ software (1.52p and 1.53f51)	Schindelin et al.^6^	https://imagej.net/software/fiji/
R (v 4.1.2)	The R Foundation	https://www.r-project.org/
RStudio Desktop	RStudio	https://www.rstudio.com/products/rstudio/
Bioconductor (v 3.14)	The Bioconductor Project	https://bioconductor.org/
Cell Ranger (v 3.1.0)	10x Genomics	https://support.10xgenomics.com/single-cell-gene-expression/software/downloads/latest
DropletUtils (v 1.14)	Lun et al.^7^	https://bioconductor.org/packages/release/bioc/html/DropletUtils.html
scater (v 1.14.6)	McCarthy et al.^8^	https://bioconductor.org/packages/release/bioc/html/scater.html
scuttle (v 1.8.0)	McCarthy et al.^8^	https://bioconductor.org/packages/release/bioc/html/scuttle.html
batchelor (v 1.2.4)	Haghverdi et al.^9^	https://bioconductor.org/packages/release/bioc/html/batchelor.html
scran (v 1.22)	Lun et al.^10^	https://bioconductor.org/packages/release/bioc/html/scran.html
Rtsne (v 0.16)	CRAN	https://cran.r-project.org/web/packages/Rtsne/index.html
igraph (v 1.3.5)	CRAN	https://cran.r-project.org/web/packages/igraph/index.html
ScDblFinder	Germain et al.^11^	https://bioconductor.org/packages/release/bioc/html/scDblFinder.html
PANTHER	Mi et al.^12^	http://www.pantherdb.org
AmiGO (v 2.5.13)	Carbon et al.^13^	http://amigo.geneontology.org/amigo

**Other**		

Stereoscope	Leica M80	N/A
Petri dish	Cellstar	N/A
1.5 mL Eppendorf tubes	Eppendorf	N/A
Water bath	Grant	N/A
Centrifuge	Rotina 380R	N/A
0.2 mL PCR tubes	Applied Biosystems by Life Technologies	Tubes (N8010580) and Lids (N8010535)
Heating shaker	Grant-Bio	PSC24
Heating block	Grant-Bio	QBT4
SimpliAmpTM Thermal Cycler	Applied Biosystems by Life Technologies	A24812
Stericup filter	Millipore	N/A
Steritop filter	Millipore	N/A
6-well and 24-well plates	Corning Incorporated	N/A
T25 and T75 flasks	Corning	N/A
Cryovials	Thermo Scientific	N/A
Brightfield microscope	Axio Observer, Zeiss	N/A

## Materials and equipment

**Table:** AGM cell dissociation media

Reagent	Stock concentration	Final concentration	Amount
Collagenase type 1	2.5% (w/v = 1 g/40 mL PBS)	0.125% (w/v)	10 μL (1:20 from stock)
PBS/10%FCS/1%PS	N/A	N/A	190 μL
Total	N/A	N/A	200 μL

Stock solution can be stored in Eppendorf aliquots at −20°C.

**Table:** MSC culture media

Reagent	Final concentration	Amount
heat-inactivated FCS	15%	30 mL
αMEM	35%	70 mL
PS	1%	2 mL
Glutamax	1%	2 mL
β-mercaptoethanol	0.02%	40 μL
MyeloCult	N/A	Up to 200 mL (96 mL)
Total	N/A	200 mL

The working solution can be stored at 4°C for 1 month.

**Table:** Osteogenic differentiation media

Reagent	Stock concentration	Final concentration	Amount
Fetal Calf Serum (FCS)	N/A	10%	10 mL
Penicillin-Streptomycin (PS)	N/A	1%	1 mL
L-ascorbic acid-2-phosphate (L-AA)	5 mM	50 μM	1 mL
β glycerophosphate disodium pentahydrate (β-GP)	1 M in DMEM	10 mM	1 mL
Dexamethasone	1 mM in DMSO	100 nM	10 μL
Gibco Dulbecco’s Modified Eagle Medium (DMEM) with Glutamax	N/A	N/A	Up to 100 mL (87 mL)
Total	N/A	N/A	100 mL

The working solution can be stored at 4°C for 1 month.

**Table:** Osteogenic control media

Reagent	Stock concentration	Final concentration	Amount
Fetal Calf Serum (FCS)	N/A	10%	10 mL
Penicillin-Streptomycin (PS)	N/A	1%	1 mL
Gibco Dulbecco’s Modified Eagle Medium (DMEM) with Glutamax	N/A	N/A	Up to 100 mL (89 mL)
Total	N/A	N/A	100 mL

The working solution can be stored at 4°C for 1 month.

**Table:** Alizarin Red Solution

Reagent	Final concentration	Amount
Alizarin red S	0.02 g/mL	2 g
Milli-Q water	N/A	100 mL
Total	N/A	100 mL

The working solution can be stored at room temperature (20°C–22°C) for 1 month and should be protected from light.

## Step-by-step method details

### Cell preparation

**Timing: 4 h**

Mouse (*Mus musculus*) embryos at day 11 of development (E11; 42–45 somite pairs), from wild type (WT, PDGRFβ+/+) and knockout (KO, PDGRFβ−/−) strains obtained by breeding heterozygous PDGRFβ+/− adults in a C57BL/6J background were used.^5^

1. Embryo dissection.
  a. Prepare a 2.5% w/v collagenase type 1 stock solution by adding 1 g of collagenase type 1–40 mL of sterile PBS and filter using a large syringe with a 0.45 μm filter.
    ***Note:*** Aliquots of the collagenase type I stock solution can be stored in Eppendorf tubes at −20°C at long-term and thawed at room temperature (20°C–22°C) or for few minutes in a water bath at 37°C on the day of the experiment.
  b. Cull pregnant dams and harvest the embryos on ice, in cold filtered PBS buffer solution enriched with 10% fetal calf serum (FCS), and 1% penicillin/streptomycin (PS), henceforth referred to as PBS/FCS/PS.
    ***Note:*** PBS/FCS/PS working solution should be stored at 4°C and used within 1 week.
  c. Dissect the embryos right after in a petri-dish with PBS/FCS/PS under a stereoscope using a pair of scissors and tweezers as previously described.^14^
    ***Note:*** The time to dissect can vary between scientists and thus all embryos should be kept on ice prior dissection.
  d. Ensure that the embryos used are at the desired stage of development by counting the somite pairs.
  e. Transfer a piece of tissue from each embryo (usually the yolk sac or the head) in an empty labeled 1.5 mL Eppendorf tube and proceed with genotyping immediately (protocol described in steps 3 and 4 below).
    ***Note:*** These tubes can be frozen at −20°C if not used on the same day. If cells are used for scRNA-seq, genotyping should be done immediately, in parallel with tissue dissection and thus, this may require 2 scientists. If AGM cells are used to derive MSCs, genotyping can be done after cells are seeded the same day or after.

2. Embryonic cell dissociation.
  a. Dissect single AGMs and transfer them in 1.5 mL Eppendorf tubes (1 AGM/tube), containing 190 μL of PBS/FCS/PS.
  b. Add 10 μL of collagenase type I from the stock solution (1:20, final 0.125% w/v) to each Eppendorf and incubate in a water bath at 37°C for 45 min.
    **CRITICAL:** Do not leave the tissue for longer than 45 min since it may be detrimental to cell viability.
  c. At room temperature (20°C–22°C), mechanically disrupt the remaining tissue by gently pipetting up and down using a P200 pipette. Repeat until the tissue is not visible anymore.
  d. Wash the cells by adding 1 mL of cold PBS/FCS/PS in each tube.
  e. Centrifuge for 10 min, at 2,000 rpm and 4°C (pre-cooled).
  f. Discard the supernatant and resuspend the cell pellet in either PBS/FCS/PS for library preparation or MSC media for culture.
3. DNA extraction.
  a. Add 100 μL of Extraction Solution and 25 μL of Tissue Preparation Solution to each sample.
  b. Incubate and shake all samples in a pre-heated shaker at 55°C for 10 min.
  c. Proceed to DNA denaturation by transferring tubes, without lid, to a pre-heated heating block at 95°C for 3 min.
  d. Remove tubes from the heating block and add 100 μL of Neutralization Solution B, then vortex for a few seconds.
  e. Store genomic DNA on ice for a few minutes while preparing the Polymerase Chain Reaction (PCR) mix. Alternatively, genomic DNA can be stored for the next day at 4°C or for up to a month at −20°C.

**Figure 1:**
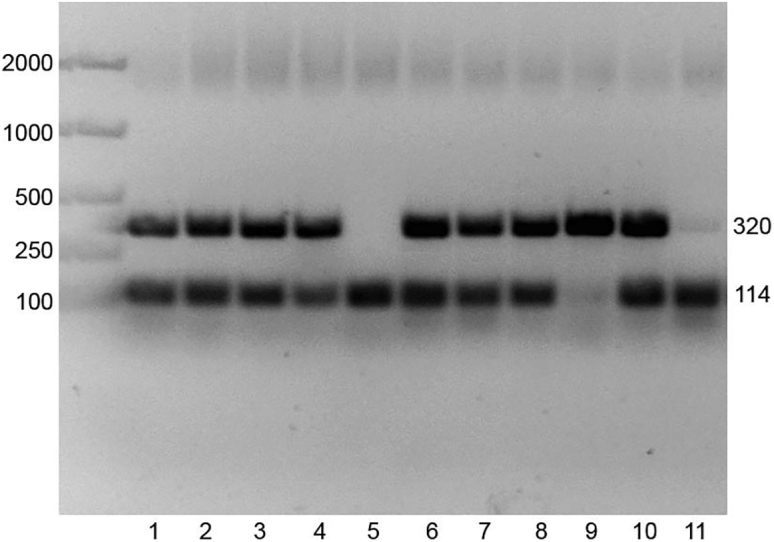
PDGFRβ-KO genotyping example The upper band represents the *ko* allele (320 bp), while the lower band represents the *wt* allele (114 bp). Samples 1, 2, 3, 4, 6, 7 and 10 are PDGFRβ +/- (heterozygous), 5 and 11 are PDGFRβ +/+ (WT) and 9 is PDGFRβ −/− (KO).
4. PCR and electrophoresis.
  a. Prepare a PCR master mix. For one sample, add 13.9 μL of autoclaved dH_2_O, then 2 μL of Coral Load Buffer 10×, 1 μL of 25 mM MgCl_2_, 1 μL of 10 mM dNTPs, 0.1 μL of each of the 3 following primers: Forward ACA ATT CCG TGC CGA GTG ACA G, WT Reverse AAA AGT ACC AGT GAA ACC TCG CTG and KO Reverse ATC AGC CTC GAC TGT GCC TTC TAG, and 0.3 μL of HotStarTaqPlus DNA polymerase (5 U/μL).
    ***Note:*** Reagents such as the Coral Load Buffer, MgCl_2_ solution and DNA polymerase are included in the Hot Start Taq Plus kit. All other reagents should be ordered separately.
  b. Dispense 18 μL of the mix to a 0.2 mL PCR tube and add 2 μL of the genomic DNA sample for a total volume of 20 μL by a short spin down.
  c. Add the PCR reaction tubes to a thermocycler and run the following program (it takes approx. 120 min): 1 cycle at 95°C for 5 min for initial DNA denaturation, 35 cycles of denaturation at 95°C for 1 min, primer annealing at 58°C for 1 min, extension at 72°C for 1 min, followed by 1 cycle for 10 min at 72°C for the final extension and hold at 10°C until stop instruction.
  d. Prepare 1.5% agarose gel in 1× TAE Buffer with SYBR®Safe DNA gel stain at 1:10 dilution.
  e. Let gel polymerize in the chemical hood at room temperature (20°C–22°C) for at least 20 min.
  f. Set up the polymerized gel in an electrophoresis tank, previously filled with fresh 1× TAE, and load a DNA molecular weight marker (20 μL of EasyLadder I).
  g. Load the samples and run the gel at 115 V and 400 mA, for 90 min.
  h. Analyze gel with a UV transilluminator and read the bands for WT at 114 bp and the KO at 320 bp (Figure 1).
    ***Note:*** The details described above are optimized for this PDGRFβ mouse strain and may differ from other murine strains.

### Single-cell RNA-sequencing data analysis

**Timing: days to weeks**

This major step describes an end-to-end analysis of scRNA-seq data, to understand the changes occurring in mesenchymal stem/stromal cells (MSCs) in the PDGFRβ-KO AGM at a single-cell resolution. The analysis steps broadly correspond with the OSCA Bioconductor workflow, based on the scran^10^ and scater^8^ Bioconductor packages^15^; similar steps may be carried out via the Seurat Bioconductor package.^16^ Code examples generally refer to a single sample (which we refer to as ‘sce_wt’); the analysis steps are almost identical for both WT and KO samples.

**Figure 2:**
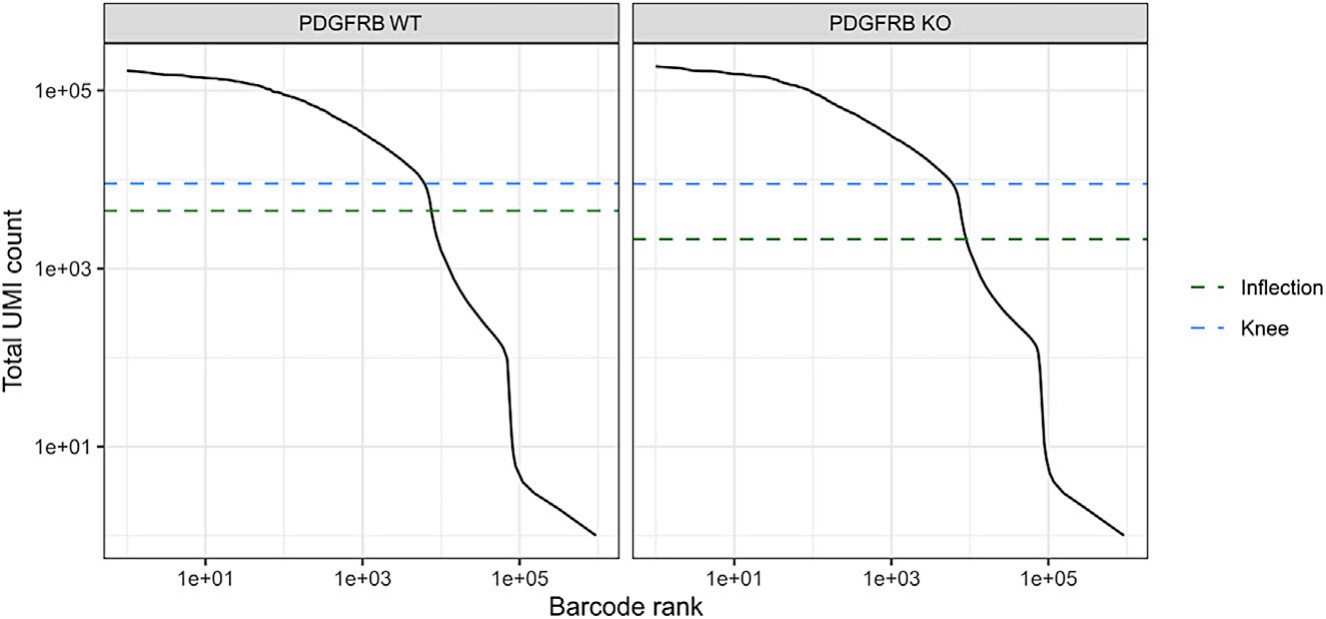
Barcode rank plots for WT and KO samples Barcode rank plots show the distribution of barcode counts and which barcodes were inferred to be associated with cells. The inflection and knee points correspond to transitions in the distribution of barcode counts, reflecting the difference between good quality droplets with many associated barcodes, and poor quality droplets, or those containing only ambient RNA. The plots should show a clear separation of these two groups of droplets, with a population of good quality droplets with high UMI counts above the knee point, followed by a sharp decrease in the number of UMIs associated with droplets.

**Figure 3:**
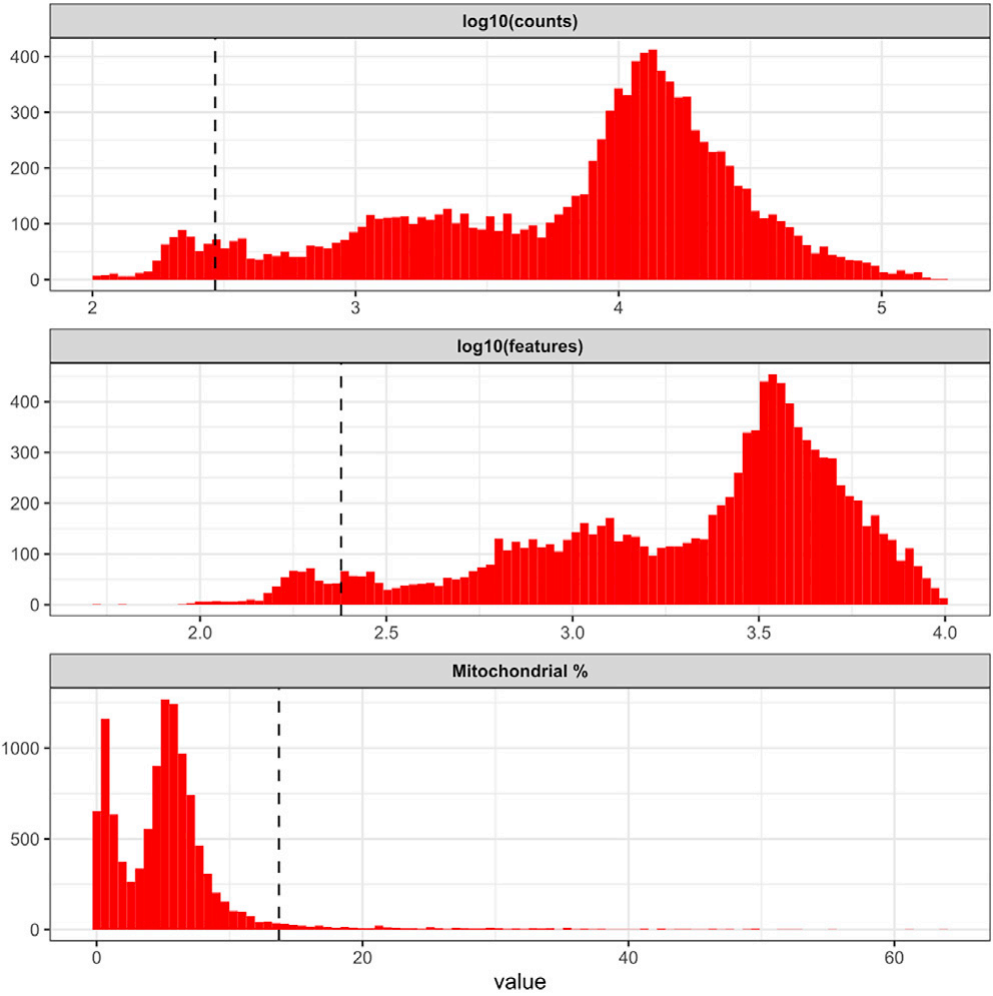
Cell-based quality control metrics Histograms illustrating three cell-based quality control metrics. Dashed lines indicate computed thresholds 3 MADs away from the median: any cell with any quality metric more extreme than 3 MADs away from the median is removed from downstream analysis.

**Figure 4:**
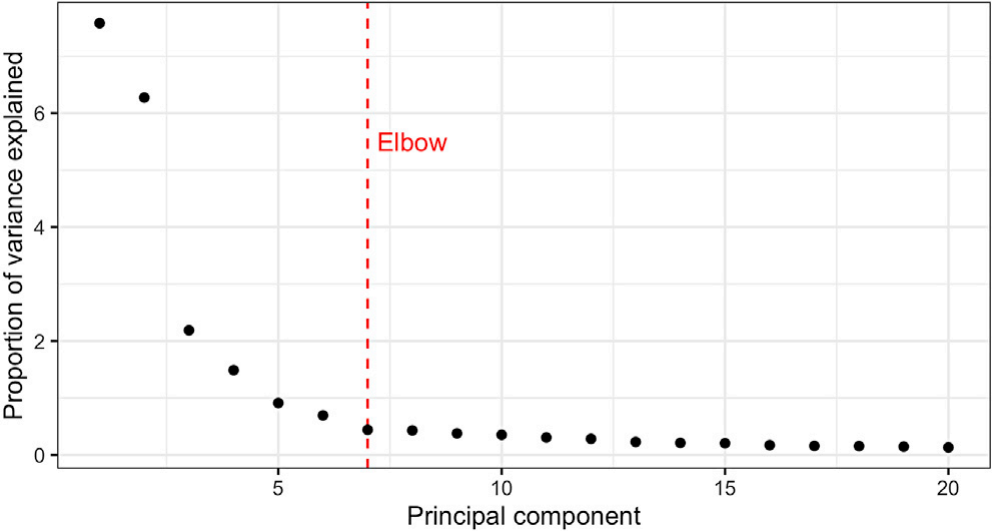
PCA scree plot PCA scree plot illustrating the proportion of variance explained by each principal component. The red line indicates an ‘elbow’ point beyond which retaining additional principal components for downstream analysis provides little additional information.

**Figure 5:**
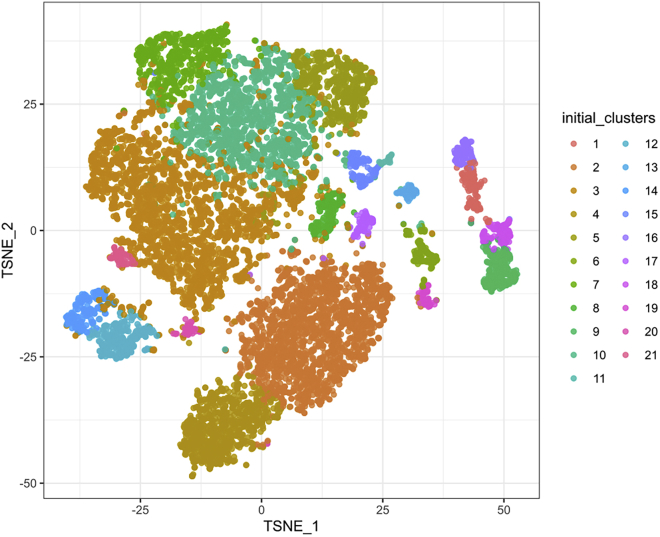
Initial WT cell clustering An initial cell clustering for the WT sample is computed by the Walktrap algorithm and used as a basis for cell type annotation.

**Figure 6:**
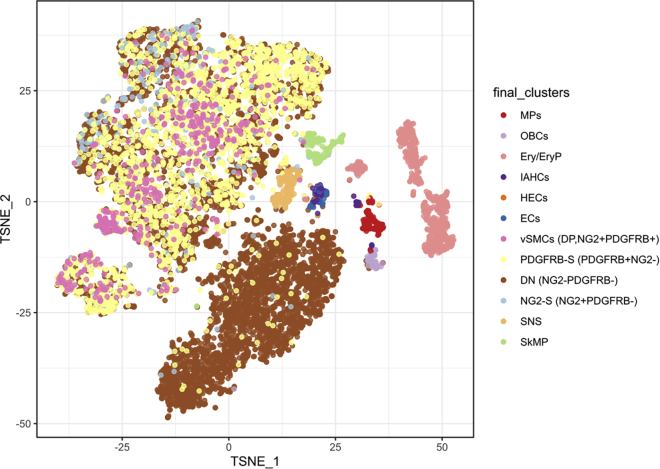
Final WT cell type annotations Following cell type annotation, the number of cell clusters is reduced and mapped to biologically relevant cell types.

**Figure 7:**
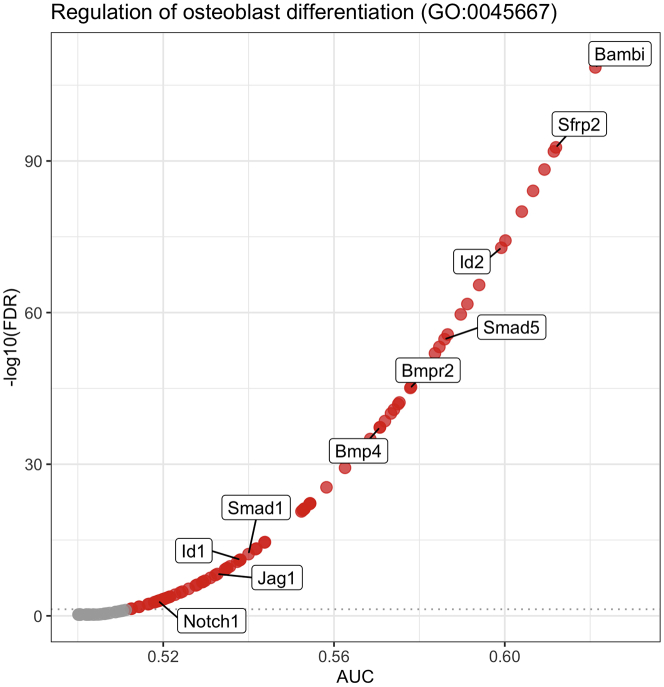
Scatterplot of effect size (reported as area under the ROC curve, AUC) vs significance, for genes associated with the GO term ‘Regulation of osteoblast differentiation’ (GO:0045667) This GO term is significantly overrepresented in genes significantly downregulated in the PDGFRβ-KO niche.^1^ Significantly downregulated genes are plotted in red and selected genes are labeled.

5. Library preparation for single-cell analysis.
  a. Prepare dissociated AGMs in a single-cell suspension as mentioned above to be processed for sample loading and library preparation as described in the protocol “Chromium Next GEM Single Cell 3′ Reagent Kits User Guide (v3.1 Chemistry)” by 10x Genomics.
  b. Load 7 × 10^3^ cells in the Chromium Next Gem Chip G, viable cells are counted in a 1:1 trypan blue ratio (cell range set for 8–11 μm) to calculate the volume of cell suspension required.
    ***Note:*** A table is provided in the 10x Genomics protocol to cross cell stock concentration (cell/μL) and the desired targeted cell recovery (for example 7 × 10^3^ cells).
  c. Single-cell libraries are to be obtained according to the manufacturer’s protocol which consist of barcoding, amplifying cDNA, gel-emulsion droplets (GEM) generation and cDNA amplification and quantification, detailed here: “Chromium Next GEM Single Cell 3′ Reagent Kits User Guide (v3.1 Chemistry)”.
  d. Quantify RNA concentration and the quality of the libraries.
  e. Send your libraries to sequencing.
6. Generating single-cell count matrices.
  a. Transfer your sequencing data from the sequencing facility to the computer where the data will be processed.
    ***Note:*** The exact steps for data transfer will vary, depending on your sequencing provider and your local compute infrastructure.
  b. At the command line, use 10x Genomics Cell Ranger to generate single cell feature counts for each sample separately, running “cellranger count” with the reference dataset previously downloaded.
    ***Note:*** Here, we used 10x Genomics reference mm10/GRCm38–3.0.0. The ‘id’ parameter can be specified to label the sample (eg.: as WT or KO).
    $ cellranger count --id=SAMPLE_ID
         --transcriptome=/path/to/reference/refdata-cellranger-mm10-3.0.0
         --fastqs=/path/to/sample/fastqs

7. Import and filter single-cell data.
  a. Download the ‘raw_feature_bc_matrix’ directory output from cellranger count for each sample: this will contain three files: barcodes.tsv.gz, features.tsv.gz and matrix.mtx.gz.
  b. In R, use read10xCounts() from the DropletUtils Bioconductor package^7^ to import the Cell Ranger output into R in the SingleCellExperiment format.
    #import cell ranger output and assign to ‘sce_wt’ object
    > sce_wt <- read10xCounts("/path/to/raw_feature_bc_matrix",col.names=T)
  c. Generate barcode rank plots (Figure 2) to monitor the distribution of barcode counts, then run emptyDrops() to identify empty droplets. Remove cells predicted to contain only ambient RNA, using the default false discovery rate (FDR) of 0.1%.
    #generate barcode rank plot for ‘sce_wt’ object
    > bcrank_wt <- barcodeRanks(counts(sce_wt))
    > uniq_wt <- !duplicated(bcrank_wt$rank)
    > plot(bcrank_wt$rank[uniq_wt],bcrank_wt$total[uniq_wt],
      log="xy",xlab="Rank",ylab="Total UMI count",
      cex.lab=1.2)
    > abline(h=metadata(bcrank_wt)$inflection,
     col="darkgreen",lty=2)
    > abline(h=metadata(bcrank_wt)$knee,
     col="dodgerblue",lty=2)
    > legend("bottomleft",legend=c("Inflection","Knee"),
    col=c("darkgreen","dodgerblue"),lty=2,cex=1.2)
    #remove cells with only ambient RNA via emptydrops
    > e.out_wt <- emptyDrops(counts(sce_wt))
    > sce_wt <- sce_wt[,which(e.out_wt$FDR<=0.001)]
    ***Note:*** As of v3, Cell Ranger implements a version of the EmptyDrops algorithm that provides similarly filtered barcode matrices, in the ‘filtered_feature_bc_matrix’ directory.

8. Annotate and perform quality control on single-cell data.
  a. Identify mitochondrial genes by annotating each sample using Bioconductor’s AnnotationHub service with an appropriate reference.
    ***Note:*** Here, we used Ensembl mm38.93 for annotation, to match that used in the creation of the 10x Genomics reference.
    #import ensembl annotation
    > ensdb_mm38.93 <- AnnotationHub()[["AH64461"]]
    > chr_loc <- mapIds(ensdb_mm38.93, keys=rownames(sce_wt),
      keytype="GENEID", column="SEQNAME")
    > is.mito <- which(chr_loc=="MT")
  b. Calculate the library size, number of expressed features and percentage of mitochondrial reads for each cell using perCellQCMetrics() from the scuttle Bioconductor package: (Figure 3).
  c. Using the median absolute deviation (MAD) definition of outliers and perCellQCFilters(), remove cells with any quality metric more extreme than 3 MADs from the median.
  d. Finally, remove any cells with detectable PDGFRβ expression from the KO sample.
    #compute QC metrics for ‘sce_wt’
    > df <- perCellQCMetrics(sce_wt, subsets=list(Mito=is.mito))
    > qc <- perCellQCFilters(df,
           sub.fields=c("subsets_Mito_percent"))
    #filter out low quality cells
    > sce_wt <- sce_wt[,!qc$discard]
    #remove cells with Pdgfrb expression from KO sample only:
    > sce_ko <- sce_ko[,(counts(sce_ko)[rowData(sce_ko)$Symbol=="Pdgfrb",]==0)]

9. Normalization and feature selection.
  a. Prepare for normalizing the expression data for each sample by running scran’s quickCluster algorithm. This is intended to quickly estimate clusters of cells with distinct expression profiles.
  b. Use the resultant clusters as input to computeSumFactors(), which implements a deconvolution method^10^ for scaling normalization of sparse count data.
    ***Note:*** We specify ‘min.mean = 0.1’ in the call to computeSumFactors(), to define the minimum (library size-adjusted) average count of genes to be used for normalization. Setting this parameter avoids using very low-abundance genes for the sum factor computation: if too many genes have consistently low counts across all cells, the computed size factors may be close to zero.
  c. Run the normalization using the computed size factors with logNormCounts().
  d. Model the variance of the log-expression profile for each gene using scran’s modelGeneVarByPoisson() function. This function decomposes log-expression into technical and biological components based on a mean-variance trend corresponding to Poisson noise and utilizes the size factors computed earlier.
  e. Finally, define highly variable genes (HVGs) for each sample using getTopHVGs().
    #quickly estimate clusters of cells and compute sum factors
    > wt_clusters <- quickCluster(sce_wt, use.ranks=F,
            BSPARAM=IrlbaParam())
    > sce_wt <- computeSumFactors(sce_wt, min.mean=0.1,
            cluster=wt_clusters)
    #run the normalization
    > sce_wt <- logNormCounts(sce_wt)
    #modelling gene variation:
    > dec_pois_wt <- modelGeneVarByPoisson(sce_wt)
    #define highly variable genes:
    > hvg_pois_wt <- getTopHVGs(dec_pois_wt)
    ***Note:*** modelGeneVar() performs a similar function to modelGeneVarByPoisson(), but tends to understate biological variation in heterogeneous datasets, such as whole unfractionated AGM, as we analyze here.

10. Dimensionality reduction and visualization.
  a. Use denoisePCA() to perform dimensionality reduction via principal components analysis (PCA) to eliminate random technical noise in the data. This function utilizes both the model of gene variance and the HVGs defined previously.
    ***Note:*** In Sá da Bandeira et al., we explicitly set the number of principal components (PCs) to retain using the min.rank and max.rank parameters as shown in this code example. These parameter settings were chosen based on an exploratory PCA, followed by analysis of the resulting scree plots (example in Figure 4). The scree plot illustrates the proportion of variance explained by each PC: the number of PCs to retain is often chosen by identifying an ‘elbow’ on the plot, beyond which retaining additional PCs explains little additional variance.
    #run PCA
    > sce_wt <- denoisePCA(sce_wt,
          technical=dec_pois_wt,
          subset.row=hvg_pois_wt,
          min.rank=10, max.rank=10)
    **CRITICAL:** The same number of PCs must be retained in all samples to subsequently integrate the samples downstream (step 13), since Batchelor’s correctExperiments() function combines the dimensional reduction matrices for each sample and these are required to be the same size. Subsequently, we are required to choose a number of PCs which explain sufficient variation in all samples.
  b. Each sample may be visualized as *t*-SNE or UMAP plots via scater’s runTSNE() and runUMAP() functions; both utilize the PCA reduction just computed.
    #run TSNE, UMAP
    > sce_wt <- runTSNE(sce_wt, use_dimred="PCA")
    > sce_wt <- runUMAP(sce_wt, use_dimred="PCA")
    #plot (example for TSNE)
    > plotReducedDim(sce_wt, "TSNE")
    ***Note:*** It is often useful to compare *t*-SNE reductions of different perplexities^17^ side-by-side. Rather than using runTSNE(), it may be advantageous to run the Rtsne() function underlying runTSNE() directly, specifying the desired perplexity and storing the resulting matrix in the reducedDims slot of the SingleCellExperiment object with an appropriate name (eg “TSNE30” rather than “TSNE”).
    #manually run, store TSNE reductions of different perplexity #here, for example, perplexity=30
    > t30 <- Rtsne(reducedDim(sce_wt,"PCA"), pca=FALSE,
        perplexity=30)
    > reducedDims(sce_wt)$TSNE30 <- t30$Y

11. Clustering.
  a. Construct a shared nearest-neighbor (SNN) graph for each sample, using scran’s buildSNNGraph() function.
  b. Compute an initial clustering for each sample (Figure 5), by using the SNN graph as input to the Walktrap community finding algorithm, using the cluster_walktrap() function from the igraph R package.
    #construct SNN graph
    > g_wt <- buildSNNGraph(sce_wt, use.dimred = "PCA")
    #compute initial clustering
    > clust_wt <- igraph::cluster_walktrap(g_wt)$membership
    > sce_wt$initial_clusters <- factor(clust_wt)
    ***Note:*** In Sá da Bandeira, et al. we use the Walktrap algorithm, but the igraph package allows for the use of other community-finding algorithms, such as Louvain.^1^ It may be worthwhile experimenting with different algorithms to explore the robustness of any cell clusters.
  c. Apply doublet detection to each sample, for example via scDblFinder’s computeDoubletDensity() function, which simulates random artificial doublets from real cells and tries to identify cells whose neighborhood has a high local density of artificial doublets.
    #doublet detection
    > dbl_den_wt <- computeDoubletDensity(sce_wt,
            subset.row=hvg_pois_wt))
    > sce_wt$doubletScore <- log1p(dbl_den_wt)
    ***Note:*** It is important to make sure that doublet detection is carried out on individual samples, prior to any merging or integrating processes; by definition, doublet cells can only arise from a single library. Clusters principally driven by a population of cells with high predicted doublet scores may be discarded; otherwise, we recommend keeping all cells, retaining their doublet score metadata for examination downstream if required.

12. Cell type annotation.
  In Sá da Bandeira et al., we combined marker data computed on the clusters defined above (using scran’s scoreMarkers() function) with literature-based markers for cell type annotation.^1^ Cell type annotations were assigned either based on the clustering computed above, or by the expression of known markers, as in the following code example:
  #annotation based on cluster number
  #1: annotating cells from a single cluster
  > sce_wt$cell_type[sce_wt$initial_clusters==0] <- "Cell_type_A"
  #2: annotating cells from multiple clusters
  > sce_wt$cell_type[sce_wt$initial_clusters %in% c(1,2)] <- "Cell_type_B"
  #annotation based on positive expression of "Gene"
  > sce_wt$cell_type[logcounts(sce_wt)[rowData(sce_wt)$Symbol=="Gene",]>0] <- "Cell_type_C"
  Following annotation, cells can be grouped and visualized by cell type (Figure 6), rather than by the automated clusters defined previously (Figure 5).
  a. First define non-perivascular cell types:
    i. *Ptprc* (CD45)+: Macrophage/Macrophage Progenitor (MP) clusters are identified by strong expression of *Adgre1* (F4/80) and *Ptprc* (CD45).
    ii. *Pecam1* (CD31)+: EC/HEC/IAHC further separated by additional markers; intra-aortic hematopoietic clusters (IAHCs) are *Kit+,* and endothelial cells/hemogenic endothelial cells (EC/HECs) are *Kit- Ptprc* (CD45)- *Runx1*- and *Runx1*+ respectively.
    iii. A cluster of cells enriched in *Pf4* (also expressed by some MPs), but not expressing CD45, are annotated as Other Blood Cells (OBCs), likely platelets.
    iv. Erythroid cells and their progenitors (Ery/EryP) are marked by expression of *Gypa.*
    v. Cell clusters deriving from the sympathetic nervous system (SNS) and skeletal muscle progenitors (SkMP) are enriched in *Ngfr* and *Myod1,* respectively.
  b. Define perivascular cell types.
    i. Perivascular cell types were defined as *Cspg4+* and/or *Pdgfrb+* if they had positive log-expression of these genes. These are defined as pericytes/vascular smooth muscle cells (PC/vSMCs), also named double positive cells (DP, NG2+PDGFRβ+), and are surrounded by PDGFRβ-single (PDGFRβ-S, NG2-PDGFRβ+) and double negative cells (DN, NG2-PDGFRβ-).^1^
    ii. NG2-single cells (NG2-S, NG2+PDGFRβ-) were found around the notochord.^1^
      ***Note:*** Cell types present (especially rare cell types) may be found in different proportions, based on the specific samples sequenced. Cell types may also be combined or split based on the research question. During the annotation process, it is helpful to visualize the expression of literature-based markers, at both the cluster level and at a sample level using violin and *t*-SNE plots.

13. Merging samples.
  a. Merge the WT and KO samples using correctExperiments() from the batchelor Bioconductor package^9^; this function applies a batch correction while combining the assay data and column metadata for downstream analysis. correctExperiments() retains batch information in the batch slot and we update this to a more useful label.
    #merging WT and KO samples
    > merged <- correctExperiments(sce_wt_filt, sce_ko_filt,
            PARAM=FastMnnParam())
    > merged$batch <- factor(merged$batch)
    > levels(merged$batch) <- c("WT","KO")
  b. Apply dimensional reduction and clustering to the merged dataset, as described above (steps 10 and 11).
  c. *t*-SNE visualization of the merged dataset in conjunction with the cell type annotations defined above (step 12) should confirm a clustering of cells by cell type, rather than genotype.
14. Differential expression analysis.
  a. Use scran’s pairwiseWilcox() function to perform differential expression analysis between groups of cells in the merged SingleCellExperiment object. The function requires a vector of group assignments for the ‘group’ parameter, which is most easily specified as a combination of batch and cell type. The desired comparison can be made by specifying a vector of the relevant groups for the ‘restrict’ parameter.
    #generate batch + cell type labels
    > merged$batch_celltype <- paste(merged$batch,
          merged$cell_type, sep="_")
    #differential expression analysis
    > pww <- pairwiseWilcox(logcounts(merged),
           groups=merged$batch_celltype,
           restrict=c("WT_Cell_type_A",
             "KO_Cell_type_A"),
           gene.names=rowData(merged)$Symbol,
           direction="up")
  b. The output from pairwiseWilcox() is a list of two elements: ‘statistics’ and ‘pairs’: ‘statistics’ (itself a list of DataFrames) contains the differential expression statistics, including the AUCs (the effect size), p-values and false discovery rate (FDR) values for each gene.
    #example pairwiseWilcox() output
    > head(pww$statistics[[1]])
    DataFrame with 6 rows and 3 columns
      AUC   p.value   FDR
     <numeric> <numeric> <numeric>
    Gene1  0.606633  4.685919e-86  8.225655e-85
    Gene2  0.603982  6.134690e-82  1.039850e-80
    Gene3  0.600158  3.521710e-76  5.637097e-75
    Gene4  0.599188  9.388226e-75  1.485138e-73
    Gene5  0.594001  2.326035e-67  3.460966e-66
    Gene6  0.591236  1.402869e-63  2.013091e-62
    ***Note:*** Other statistical tests may be used (eg *t*-tests via the pairwiseTTests() function). Here, we used Wilcoxon rank sum tests since they are considered more robust to outliers and insensitive to non-normality in comparison to *t*-tests. We note, however, that the disadvantages of Wilcoxon rank sum tests compared to *t*-tests include their longer running time, and the reported effect sizes of Wilcoxon rank sum tests are less interpretable.^10^

15. Functional analysis.
  Associate significantly differentially expressed genes (DEGs) with Gene Ontology (GO) terms:
  a. Define DEGs as genes with FDR<0.05 and use their gene symbols as input to the PANTHER web resource [http://pantherdb.org/].^18^
  b. Select ‘Mus musculus’ as the organism and ‘Statistical overrepresentation test’ as the analysis, with ‘GO biological process complete’ as the annotation set.
    ***Note:*** By default, PANTHER returns only significant (FDR<0.05) biological processes.
  c. Manually curate the significantly overrepresented GO terms for terms relating to osteogenesis.
  d. Search GO terms of interest in the AmiGO web resource [http://amigo.geneontology.org/amigo], which lists genes associated with a given GO term.
16. Download the associated genes for any GO terms of interest and cross-reference these with the DEGs to find genes which contributed most strongly to these GO terms (either by significance, or by the AUC (effect size) computed by the Wilcoxon rank sum test) (Figure 7).

### Mesenchymal stem/stromal cell (MSC) derivation and culture

**Timing: days to weeks**

We developed a mesenchymal cell culture to investigate whether the osteogenic developmental potential of PDGFRβ−/− AGM-derived MSCs is impaired as suggested by our scRNA-seq data analysis *in vivo.* We first tested whether mesenchymal stem/stromal cell lines can be derived from single WT AGMs then whether MSCs can be derived in the absence of PDGFRβ. The specific steps are as follows:

17. Prepare MSC medium.
  a. Heat-inactivate the FCS for 30 min in a 56°C water bath.
  b. Mix all medium components (See table in “reagents and materials” section) in a steritop filter, connected to a stericup by a vacuum pump by adding the largest volumes first.
  c. Filter and label the solution.

***Note:*** This media has been previously described^4^ and should be stored at 4°C for 1 month.

18. Seeding of AGM cells.
  a. Pre-coat a 6-well plate with 2 mL of sterile 0.1% cold gelatin for at least 1 h at room temperature (20°C–22°C) or 10 min at 4°C.
  b. Gently aspirate out the remaining gelatin and proceed without washing.
  c. Resuspend cell pellets obtained from a single AGM in 3 mL of MSC medium. A number of 21.2 +/- 6.2 × 10^4^ cells are expected to be obtained from single E11 AGMs.^14^
  d. Seed each single suspension in one well in a pre-coated 6-well plate (=passage 0).

**Figure 8:**
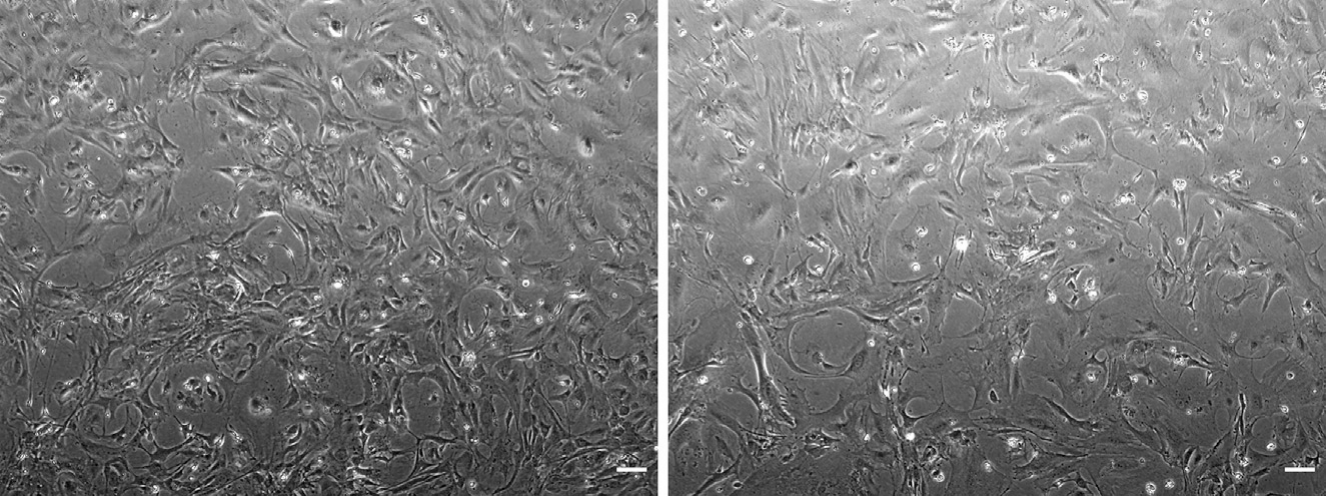
Phase-contrast images of PDGFRβ+/+ (WT, left) and PDGFRβ−/− (KO, right) single AGM-derived MSCs taken with Evos Digital Inverted Microscope Scale: 100 μm.

***Note:*** After 24 h, the cells should be completely adherent to the bottom of the well.

19. Maintenance and expansion of AGM-derived MSCs.
  a. In the first week, refresh the stromal medium only once, approx. 2–3 days after cells were first seeded.
  b. When the wells are >90% confluent (approx. 4–6 days), passage the cells from one well of a 6-well plate to one gelatin pre-coated T25 flask (=passage 1) using pre-warmed Trypsin + EDTA (0.25%) to detach the adherent cells.
  c. From here on, refresh the medium bi-weekly.
  d. When the T25 flasks are >90% confluent (approx. 1 week), passage the cells from one T25 flask to one gelatin pre-coated T75 flasks (=passage 2) using 0.25% Trypsin + EDTA to detach the adherent cells for approx. 12–15 min.
  e. When the T75 flasks are >90% confluent (approx. 1 week), expand the culture at a 1:3 ratio (from one T75 to three T75 flasks and so on) for the following weeks and passages.
    ***Note:*** When we initiate the culture with freshly harvested cells (passage 0), some MSC primary lines require 2–3 extra days to expand. This is a relatively short time and does not influence their growth or their differential potential at later stages. We were able to expand these cells and freeze/thaw them regularly for about 12 passages, independently of their genotype. However, their osteogenic potential was only tested between passage 3 and passage 6 while their hematopoiesis support was tested up to passage 12.^1^
    **CRITICAL:** Cells need to be kept in the incubator at 37°C and 5% CO_2_ at all times.
  f. After expansion, a fraction can be used for experiments and the remaining cells can be frozen and stored in liquid N_2_ or passaged.
    ***Note:*** To freeze MSCs, use around 0.5–1 × 10^6^ cells per cryovial with 90% cold FCS and 10% dimethyl sulphoxide (DMSO). To thaw cells, first prepare a 50 mL falcon tube containing 30 mL of PBS/FCS/PS. Keep the cryovial in the water bath at 37°C for a few seconds until the frozen block starts to detach, then rapidly transfer the frozen block to the 50 mL falcon tube in the tissue culture hood. Centrifuge cells at 4°C and 2,000 rpm for 5 min. Discard the supernatant, resuspend the pelleted cells with MSC medium and transfer to a gelatin pre-coated T75 flask, then incubate them at 37°C. After 24 h, renew the medium to discard the floating cells. Proceed as normal.
  g. Both WT and KO stromal cells should show a fibroblast-like morphology and resemble MSCs (Figure 8).

### Functional validation: Osteogenesis assay

**Timing: 3–4 weeks**

Mesenchymal stem/stromal cells are multipotent and therefore they are able to differentiate into bone, adipose tissue, and cartilage.^19^ Since we found a significant downregulation in osteogenic gene expression (step 14), and mesenchymal and osteogenic differentiation biological processes were significantly affected (steps 15 and 16), we performed an osteogenic assay using our expanded WT and KO-derived single AGM stromal cell cultures (steps 17–19)^1^ to validate the functional changes. To reveal calcium deposits, we used an alizarin red solution.

20. Prepare solutions.
  a. In a 100 mL flask prepare the osteogenic medium.
  b. Mix thoroughly and filter.
    ***Note:*** This media should be stored at 4°C for no longer than 1 month.
  c. In a 100 mL flask prepare the control osteogenic medium.
  d. Mix thoroughly and filter.
    ***Note:*** This media should be stored at 4°C for no longer than 1 month.
  e. In a medium Schott flask, prepare the Alizarin red solution. The pH of this solution needs to be adjusted to 4.1–4.3. Do this by adding HCl or NH_4_OH as needed.
    ***Note:*** This media should be stored at room temperature (20°C–22°C), for 1 month while protected from light covered with aluminium foil.

21. Cell differentiation assay.
  a. Seed 4 × 10^4^ MSCs per well in a 24-well plate with MSC medium.
  b. After 24 h of culture, remove the medium gently, and replace with 500 μL of either osteogenic or control media.
    ***Note:*** The plates are uncoated to avoid autofluorescence upon staining. Cells need to be at least 80%–90% confluent otherwise more time is needed to have a good cell coverage. The osteogenic medium will trigger the cells to differentiate towards the osteogenic lineage only if cells have this potential, while the control medium will not.
  c. Incubate the plate at 37°C and 5% CO2 for 21 days.
  d. Refresh the media three times every week.
22. Alizarin red staining to allow detection of calcium deposition.
  a. On day 21, discard the osteogenic and control media.
  b. Wash the wells gently with Milli-Q water to remove any media left.
  c. Fix cells by incubating the wells with 4% PFA for 10 min at room temperature (20°C–22°C).
  d. Remove the PFA and wash twice with Milli-Q water.
  e. Add 500 μL of alizarin red solution to each well, including both differentiated and control wells, for 15 min at room temperature (20°C–22°C) and in the dark.
    ***Note:*** It is important to be careful with this alizarin red solution since it easily leaves a stain mark on clothes, surfaces etc.
  f. Carefully remove alizarin red solution and wash twice with Milli-Q water.
  g. Perform a third wash with Milli-Q water but do not discard.
  h. Image the full wells with a brightfield microscope.
    ***Note:*** Stained wells can be carefully washed several times if the water is still red. Stained plates can be imaged right after the staining or kept at 4°C for several days prior imaging. In this protocol, a Zeiss Axio Observer was used and images were analyzed with Fiji/ImageJ software (v. 1.52p).

23. Brightfield image analysis.
  a. In the ZEN 2.3 Pro software, upon image acquisition, select the tiling and stitching options to generate high-resolution pictures of your full wells.
  b. Export stitched tiled images in the .czi format.
  c. In Fiji/ImageJ software, import .czi images to adjust the brightness using the same settings for all wells.
  d. Export the final image in .tiff format.
  e. Perform a qualitative observation of which MSC lines are osteogenic, if you detect alizarin red staining or low/no osteogenic, if you detect low to no staining.

***Note:*** A quantitative measure can be done in these samples by reading the plate on a spectrophotometer at a wavelength of 450 nm.

## Expected outcomes

**Figure 9:**
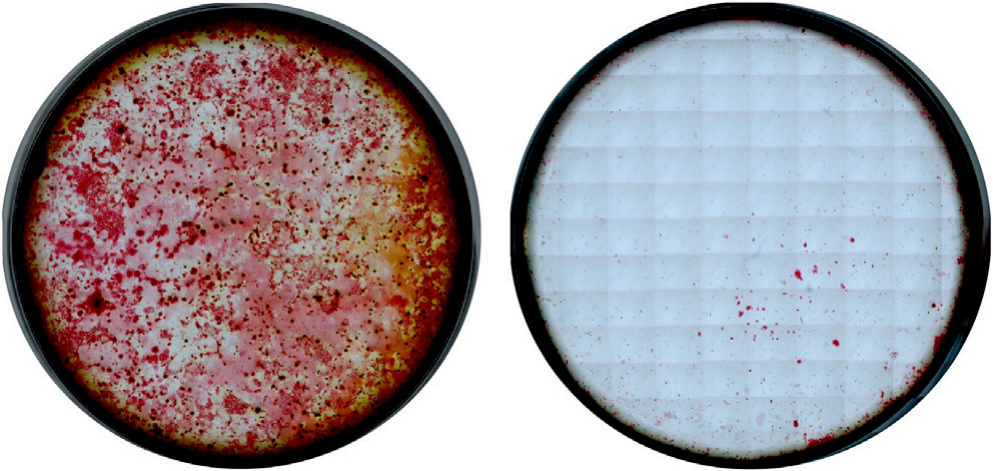
Example of wells containing PDGFRβ+/+ (WT, left) and PDGFRβ−/− (KO, right) single AGM-derived MSCs in differentiating osteogenic media and stained with alizarin red

The calcium containing cells in the differentiated culture will be stained red and can vary from light to dark red (Figure 9). Most PDGFRβ-KO stromal cell lines do not differentiate into calcium-containing cells.

## Limitations

Dissecting embryos can be challenging, and thus, this step needs to be performed by a trained scientist.

## Troubleshooting

### Problem 1

The gel doesn’t show any bands (step 4).

### Potential solution

The absence of bands may be due to a poor DNA extraction. This can be fixed by repeating the DNA extraction step followed by a new PCR and gel. Missing the polymerase from the PCR master mix could be another reason that can be fixed by repeating the PCR step.

### Problem 2

Running the same R/Bioconductor function on the same data returns different results (step 7).

### Potential solution

Many analysis functions use random processes, e.g., emptyDrops() performs Monte Carlo simulations to compute p-values, so setting a random seed via set.seed() is required to obtain reproducible results. It is therefore worthwhile testing several random seeds to ensure results are robust.

### Problem 3

Clustering my scRNA-seq data yields too many/not enough cell clusters (step 11).

### Potential solution

The choice of the connectivity parameter ‘k’ in buildSNNGraph() effectively controls the number of output clusters; smaller values correspond to more smaller and finer clusters and larger values result in fewer, more general, clusters.

### Problem 4

I am struggling to annotate cell types based on the computed cluster markers (step 12).

### Potential solution

Automated cell type annotation packages, e.g., SingleR,^20^ may be used to predict cell types based on reference (e.g., FACS sorted or pre-annotated single-cell) datasets. However, the cell type prediction relies on the quality of annotations and/or cell types included in the reference dataset. These methods may also struggle with cell types that express similar markers (e.g., ECs, HECs, IAHCs in this dataset).

### Problem 5

In the end of the alizarin staining, some differentiated (calcium containing) red cells detached and are now floating (step 22).

### Potential solution

Indeed, the differentiated cells detach easily and may be lost during the staining. To avoid this, do not keep the cells in differentiating media for longer than 21 days as they are not expected to differentiate further. To avoid losing stained cells, each liquid used (Milli-Q water, alizarin red, and PFA) should be added or aspirated by using a P200 pipette with tips kept against the walls of the wells. This careful handling prevents any direct pressure on the cells or any contact with the cells.

## Resource availability

### Lead contact

Further information and requests for resources and reagents should be directed to and will be fulfilled by the lead contact [Mihaela Crisan] (Mihaela.crisan@ed.ac.uk).

### Materials availability

This study did not generate new unique reagents.

## Data Availability

Original scRNA-seq data is available at NCBI GEO accession number GSE162103.

## References

[1] Sá da Bandeira D., Kilpatrick A.M., Marques M., Gomez-Salazar M., Ventura T., Gonzalez Z.N., Stefancova D., Rossi F., Vermeren M., Vink C.S. (2022). PDGFRbeta(+) cells play a dual role as hematopoietic precursors and niche cells during mouse ontogeny. Cell Rep..

[2] Medvinsky A., Dzierzak E. (1996). Definitive hematopoiesis is autonomously initiated by the AGM region. Cell.

[3] Mendes S.C., Robin C., Dzierzak E. (2005). Mesenchymal progenitor cells localize within hematopoietic sites throughout ontogeny. Development.

[4] Oostendorp R.A.J., Harvey K.N., Kusadasi N., de Bruijn M.F.T.R., Saris C., Ploemacher R.E., Medvinsky A.L., Dzierzak E.A. (2002). Stromal cell lines from mouse aorta-gonads-mesonephros subregions are potent supporters of hematopoietic stem cell activity. Blood.

[5] Soriano P. (1994). Abnormal kidney development and hematological disorders in Pdgf beta-receptor mutant mice. Genes Dev..

[6] Schindelin J., Arganda-Carreras I., Frise E., Kaynig V., Longair M., Pietzsch T., Preibisch S., Rueden C., Saalfeld S., Schmid B. (2012). Fiji: an open-source platform for biological-image analysis. Nat. Methods.

[7] Lun A.T.L., Riesenfeld S., Andrews T., Dao T.P., Gomes T., Marioni J.C., Participants in the 1st Human Cell Atlas Jamboree (2019). EmptyDrops: distinguishing cells from empty droplets in droplet-based single-cell RNA sequencing data. Genome Biol..

[8] McCarthy D.J., Campbell K.R., Lun A.T.L., Wills Q.F. (2017). Scater: pre-processing, quality control, normalization and visualization of single-cell RNA-seq data in R. Bioinformatics.

[9] Haghverdi L., Lun A.T.L., Morgan M.D., Marioni J.C. (2018). Batch effects in single-cell RNA-sequencing data are corrected by matching mutual nearest neighbors. Nat. Biotechnol..

[10] Lun A.T.L., McCarthy D.J., Marioni J.C. (2016). A step-by-step workflow for low-level analysis of single-cell RNA-seq data with Bioconductor. F1000Research.

[11] Germain P.L., Lun A., Garcia Meixide C., Macnair W., Robinson M.D. (2021). Doublet identification in single-cell sequencing data using scDblFinder. F1000Research.

[12] Mi H., Muruganujan A., Casagrande J.T., Thomas P.D. (2013). Large-scale gene function analysis with the PANTHER classification system. Nat. Protoc..

[13] Carbon S., Ireland A., Mungall C.J., Shu S., Marshall B., Lewis S., AmiGO Hub, Web Presence Working Group (2009). AmiGO: online access to ontology and annotation data. Bioinformatics.

[14] Dzierzak E., de Bruijn M. (2002). Isolation and analysis of hematopoietic stem cells from mouse embryos. Methods Mol. Med..

[15] Amezquita R.A., Lun A.T.L., Becht E., Carey V.J., Carpp L.N., Geistlinger L., Marini F., Rue-Albrecht K., Risso D., Soneson C. (2020). Orchestrating single-cell analysis with Bioconductor. Nat. Methods.

[16] Hao Y., Hao S., Andersen-Nissen E., Mauck W.M., Zheng S., Butler A., Lee M.J., Wilk A.J., Darby C., Zager M. (2021). Integrated analysis of multimodal single-cell data. Cell.

[17] van der Maaten L., Hinton G. (2008). Visualizing data using t-SNE. J. Mach. Learn. Res..

[18] Mi H., Muruganujan A., Huang X., Ebert D., Mills C., Guo X., Thomas P.D. (2019). Protocol update for large-scale genome and gene function analysis with the PANTHER classification system (v.14.0). Nat. Protoc..

[19] Gomez-Salazar M., Gonzalez-Galofre Z.N., Casamitjana J., Crisan M., James A.W., Péault B. (2020). Five decades later, are mesenchymal stem cells still relevant?. Front. Bioeng. Biotechnol..

[20] Aran D., Looney A.P., Liu L., Wu E., Fong V., Hsu A., Chak S., Naikawadi R.P., Wolters P.J., Abate A.R. (2019). Reference-based analysis of lung single-cell sequencing reveals a transitional profibrotic macrophage. Nat. Immunol..

